# Lipid profile analysis of donkey milk during the lactation

**DOI:** 10.3389/fnut.2025.1662407

**Published:** 2025-09-19

**Authors:** Xinyi Du, Zongjie Ma, Changfa Wang, Miaomiao Zhou

**Affiliations:** College of Agriculture and Biology, Liaocheng Research Institute of Donkey High-Efficiency Breeding and Ecological Feeding, Liaocheng University, Liaocheng, China

**Keywords:** lipids, fatty acids, milk, donkey, lactation stage

## Abstract

**Introduction:**

From ancient times, donkey milk (DM) has attracted much attention due to its particular properties. Donkey milk is characterized by a high level of lactose and low levels of protein and fat. However, studies investigating lipid changes in DM during lactation are limited.

**Methods:**

The lipid profile of DM at different lactation stages was analyzed using lipidomics in this study. Milk samples were collected from six lactating Dezhou donkeys on days 1, 30, 60, 90, 120, 150, and 180 of lactation.

**Results:**

A total of 1,552 lipids were identified, belonging to 5 major categories and 21 subclasses. Glycerophospholipids (GP, 62.11%) and sphingolipids (SP, 22.94%) were the predominant categories. At the subclass level, phosphatidylcholine (PC, 27.26%), phosphatidylethanolamine (PE, 17.59%), and phosphatidylserine (PS, 6.77%) were the most abundant lipids. Significant variations in the levels of GP, SP, glycerolipids (GL), and free fatty acids (FA) were observed across lactation stages. Colostrum was rich in GP and SP, whereas mature milk contained more GL. Regarding FA, mature milk had a higher content of linolenic acid than colostrum, while colostrum was richer in carnitines. Fourteen lipids were identified as potential biomarkers for distinguishing between DM from different lactation stages.

**Discussion:**

These results indicate that the lactation stage significantly affects the lipid composition of DM. This study provides a detailed lipidomic profile of DM, which will facilitate its further development and utilization.

## 1 Introduction

From ancient times, donkey milk (DM) has attracted much attention due to its particular properties. Donkey milk is characterized by a high level of lactose and low levels of protein and fat (1, 2). Milk lipids are important sources of energy for infants (3, 4). Lipids also have additional physiological roles, including cell membrane composition and involvement in cell signaling (5). The fat content of DM ranges from 0.3 to 1.8%, which is lower than that of buffaloes, cows, goats, sheep, and yaks (4, 6, 7). Owing to its low fat content, DM can be part of a low-calorie diet or act as a source of food for the elderly (8, 9). Moreover, similar to human milk, DM is rich in unsaturated fatty acids (UFAs) and essential fatty acids (FAs) (4, 6). DM has low fat content and a favorable FA composition, making it a potential functional food ingredient (9). Research has shown that DM can modulate glucose and lipid metabolism, which enhance the antioxidant and anti-inflammatory abilities in animals (7, 10).

Milk fat is a highly variable milk component, and its composition varies greatly among different animals (4); it is also influenced by many other factors, such as diet, individual differences, daily rhythms, lactation stage, and season (11, 12). For example, increasing the proportion of fresh herbage in the diet increased the milk fat and polyunsaturated fatty acid (PUFA) contents (11). Furthermore, Ren et al. (13) reported that dietary roughage significantly affected the lipid and volatile organic compound contents of DM. The fat content and FA composition also vary with lactation stage. Some studies have shown that the content of milk fat and saturated FAs (SFAs) decreases during lactation, whereas the content of UFAs increases (14, 15). Season also has an effect on milk fat composition. In winter, DM had a lower percentage of short-chain SFAs but higher levels of long-chain SFAs and monounsaturated FAs (MUFAs); in the cool season, it contained lower C18:0 and higher palmitoleic, oleic, and vaccenic acids (15).

Lipids can be classified into eight main categories: glycerolipid (GL), glycerophospholipid (GP), sphingolipid (SP), sterol lipid (ST), fatty acyl (FA), polyketide (PK), prenolipid (PR), and saccharolipid (SL) (44). FA, GL, GP, and SP are the primary lipid categories in milk (3). The compositions of GL, FA, GP, and SP in DM have been previously investigated (8, 16–18); however, studies investigating lipid changes in DM during lactation are limited. Therefore, this study employed lipidomics technology to analyze differential lipids in DM across different lactation periods.

## 2 Materials and methods

### 2.1 Milk samples collection

Donkey milk was collected from six healthy lactating Dezhou donkeys at different days in milk (DIM): 1 (A), 30 (B), 60 (C), 90 (D), 120 (E), 150 (F), and 180 (G). The lactating donkeys were from a farm in Liaocheng City, China. The donkeys had free access to water and were fed the same diet consisting of grass hay (available *ad libitum*) supplemented with 2 kg of concentrate per donkey per day. The donkeys were milked by hand twice daily at 11 am and 3 pm. The foals were separated from their dams for 4 h prior to milking. The collected milk samples were rapidly frozen in liquid nitrogen and stored at −80 °C until analysis.

### 2.2 Lipid extraction

A volume of 100 μl of milk sample was mixed with 0.75 ml of methanol in a glass tube and vortexed. Then, 2.5 ml of methyl tertiary-butyl ether (MTBE) was added, and the mixture was incubated in a shaker for 1 h at room temperature. Afterwards, 0.625 ml of MS-grade water was added. After 10 min of incubation, the sample was centrifuged for 10 min at 1,000 × g. The upper (organic) phase was collected, and the lower phase was re-extracted using the method described above. The combined organic phases were dried and then reconstituted in 100 μl of isopropanol (45). Finally, the extracts were analyzed by liquid chromatography-tandem mass spectrometry (LC-MS/MS).

### 2.3 UHPLC-MS/MS analysis

The Vanquish UHPLC system (Thermo Fisher, Germany) coupled with an Orbitrap Q Exactive™ HF mass spectrometer (Thermo Fisher, Germany) was used for UHPLC-MS/MS analyses. Separation was achieved using a Thermo Accucore C30 column (150 × 2.1 mm, 2.6 μm) maintained at 40 °C with a flow rate of 0.35 ml/min. The mobile phase consisted of (A) acetonitrile/water (60:40, v/v) containing 10 mM ammonium acetate and 0.1% formic acid, and (B) acetonitrile/isopropanol (10:90, v/v) containing 10 mM ammonium acetate and 0.1% formic acid. The following gradient program was applied: 70% A (2 min), 57% A (5 min), 45% A (5.1 min), 30% A (11 min), 1% A (16 min), and 70% A (18.1 min).

The Q Exactive™ HF mass spectrometer was operated in both positive and negative ionization modes with the following parameters: sheath gas flow rate, 40 psi; sweep gas flow rate, 10 L/min; auxiliary gas flow rate, 10 L/min (positive mode) or 7 L/min (negative mode); spray voltage, 3.5 kV; capillary temperature, 320 °C; heater temperature, 350 °C; S-lens RF level, 50; scan range, *m*/*z* 114–1,700; automatic gain control target, 3e6; normalized collision energy, 22, 24, 28 eV; injection time, 100 ms; isolation window, 1 *m*/*z*; automatic gaincontrol target for MS^2^, 2e5; dynamic exclusion, 6 s.

### 2.4 Data search

The raw data files generated by UHPLC-MS/MS were processed using Compound Discoverer 3.1 (CD3.1, Thermo Fisher) to perform peak alignment, peak picking, and quantitation for each metabolite. The main parameters were set as follows: retention time tolerance, 0.2 min; mass tolerance, 5 ppm; signal intensity tolerance, 30%; signal-to-noise ratio, 3; and minimum intensity, 100,000. Subsequently, the peak intensities were normalized to the total spectral intensity. The normalized data was used to predict the molecular formula based on additive ions, molecular ion peaks and fragment ions. And then peaks were matched with the Lipidmaps and Lipidblast database to obtained the accurate qualitative and relative quantitative results. Statistical analyses were performed using R (version 3.4.3) and Python (version 2.7.6). When the data were not normally distributed, normal transformations were attempted using the area normalization method.

### 2.5 Data analysis

Principal component analysis (PCA) and partial least squares-discriminant analysis (PLS-DA) were performed using MetaX. The statistical significance (*P*-value) was calculated using a *t*-test. Metabolites were considered differential if they met the following criteria: VIP > 1, *P* < 0.05, and [fold change (FC) ≥2 or FC ≤ 0.5]. For clustering heatmaps, the data were normalized to *z*-scores based on the intensity areas of the differential metabolites and were visualized using the Pheatmap package in R.

Data were analyzed by one-way ANOVA in GraphPad Prism 6 software. Data are presented as mean ± SD. A statistically significant difference is defined as *P* < 0.05.

## 3 Results

### 3.1 Lipid classes of DM

**Figure 1:**
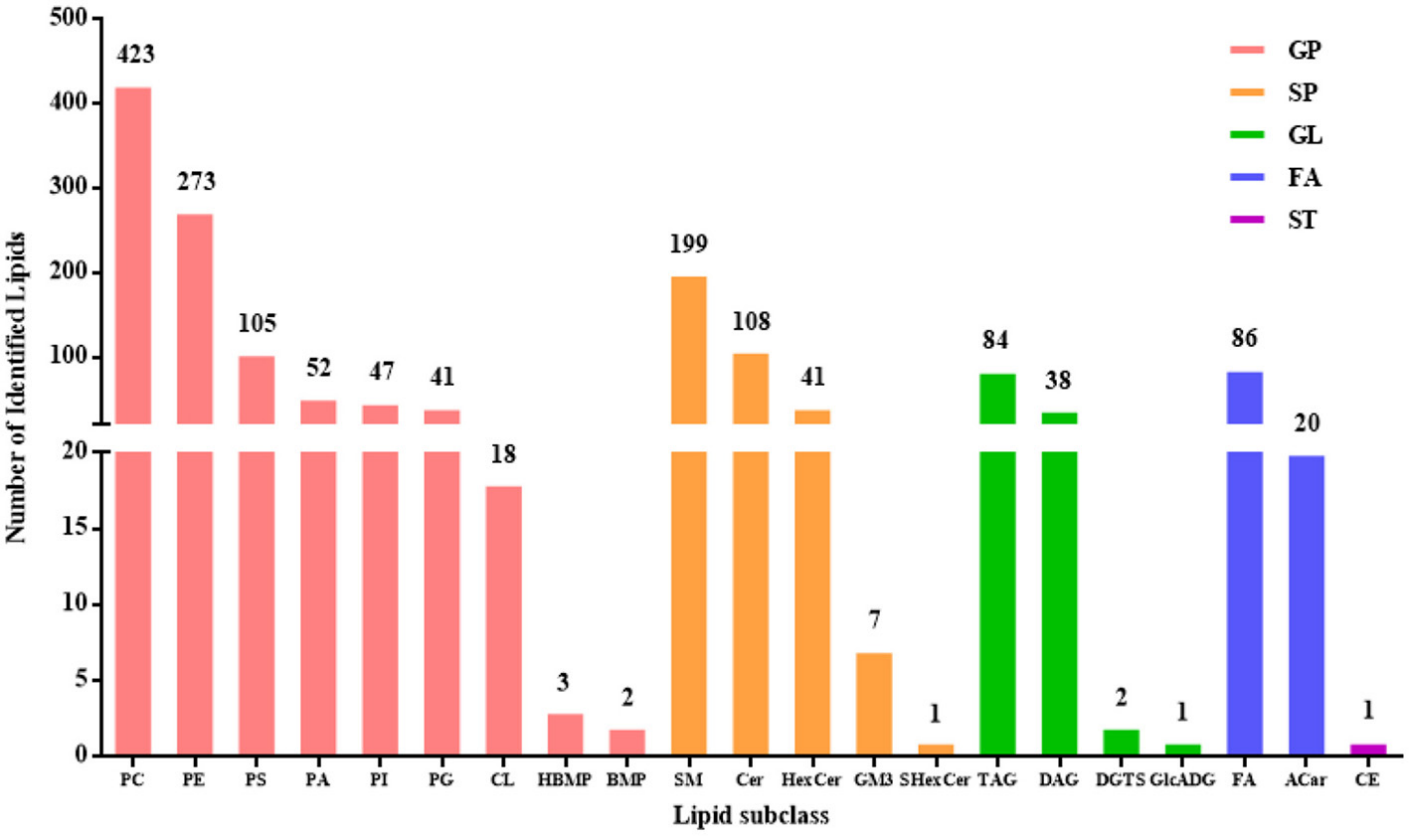
The numbers of lipids identified in DM.

A total of 1,552 lipids, spanning five categories and 21 subclasses, were detected in DM (Figure 1). The composition by category was as follows: 964 glycerophospholipids (GP, 62.11%), 356 sphingolipids (SP, 22.94%), 125 glycerolipids (GL, 8.05%), 106 fatty acyls (FA, 6.83%), and 1 sterol lipid (ST, 0.06%). The identified lipid subclasses consisted of 423 glycerophosphatidylcholine (PC) (27.26%), 273 glycerophosphatidylethanolamine (PE) (17.59%), 105 glycerophosphatidylserine (PS) (6.77%), 52 phosphatidic acid (PA) (3.35%), 47 glycerophosphatidylinositol (PI) (3.03%), 41 glycerophosphatidylglycerol (PG) (2.64%), 18 CL (1.16%), 3 hemi-bis(monoacylglycerol)phosphate (HBMP) (0.19%), 2 bis (monoacylglycerol) phosphate ester (BMP) (0.13%), 199 sphingomyelin (SM) (12.82%), 108 ceramide (Cer) (6.96%), 41 hexosylceramide (HexCer) (2.64%), 7 monosialodihexosylganglioside (GM) (0.45%), 1 sulfatides hexosyl-ceramide (SHexCer) (0.06%), 84 triacylglycerol (TAG) (5.41%), 38 diacylglycerol (DAG) (2.45%), 2 diacylglyceryl trimethylhomoserine (DGTS) (0.13%), 1 glucuronosyldiacylglycerol (GlcADG) (0.06%), 86 free fatty acid (FA) (5.54%), 20 acylcarnitine (Acar) (1.29%), and 1 cholesteryl esters (CE) (0.06%).

### 3.2 Lipid profiles of DM among different lactation stages

**Figure 2:**
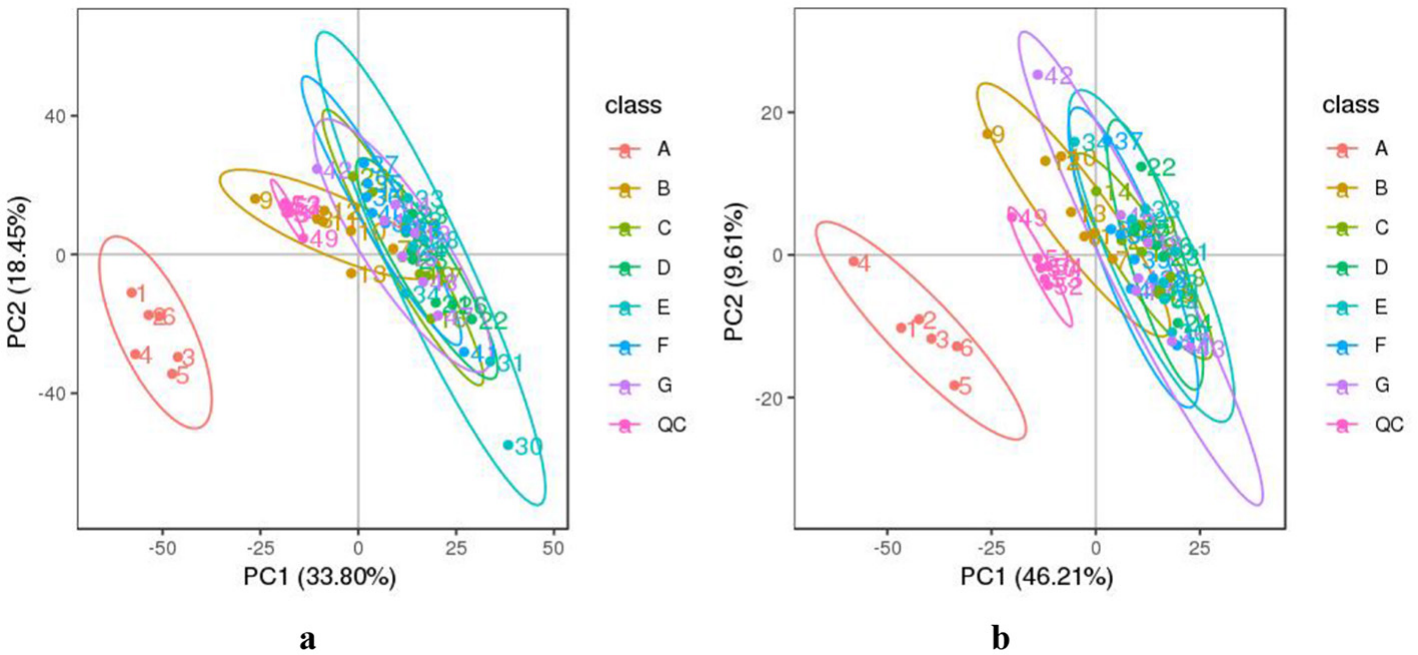
The PCA analysis of DM samples at different lactation stages. **(a)** The negative and **(b)** positive ion mode. The horizontal axis PC1 and vertical axis PC2 represent the scores of the principal components ranked first and second, respectively. Different colored dots represent samples from different groups. Ellipses represent a 95% confidence interval. QC, quality control. The milk samples were taken from six lactating donkeys at seven different lactation stages (DIM): 1 (A), 30 (B), 60 (C), 90 (D), 120 (E), 150 (F), and 180 (G).

**Figure 3:**
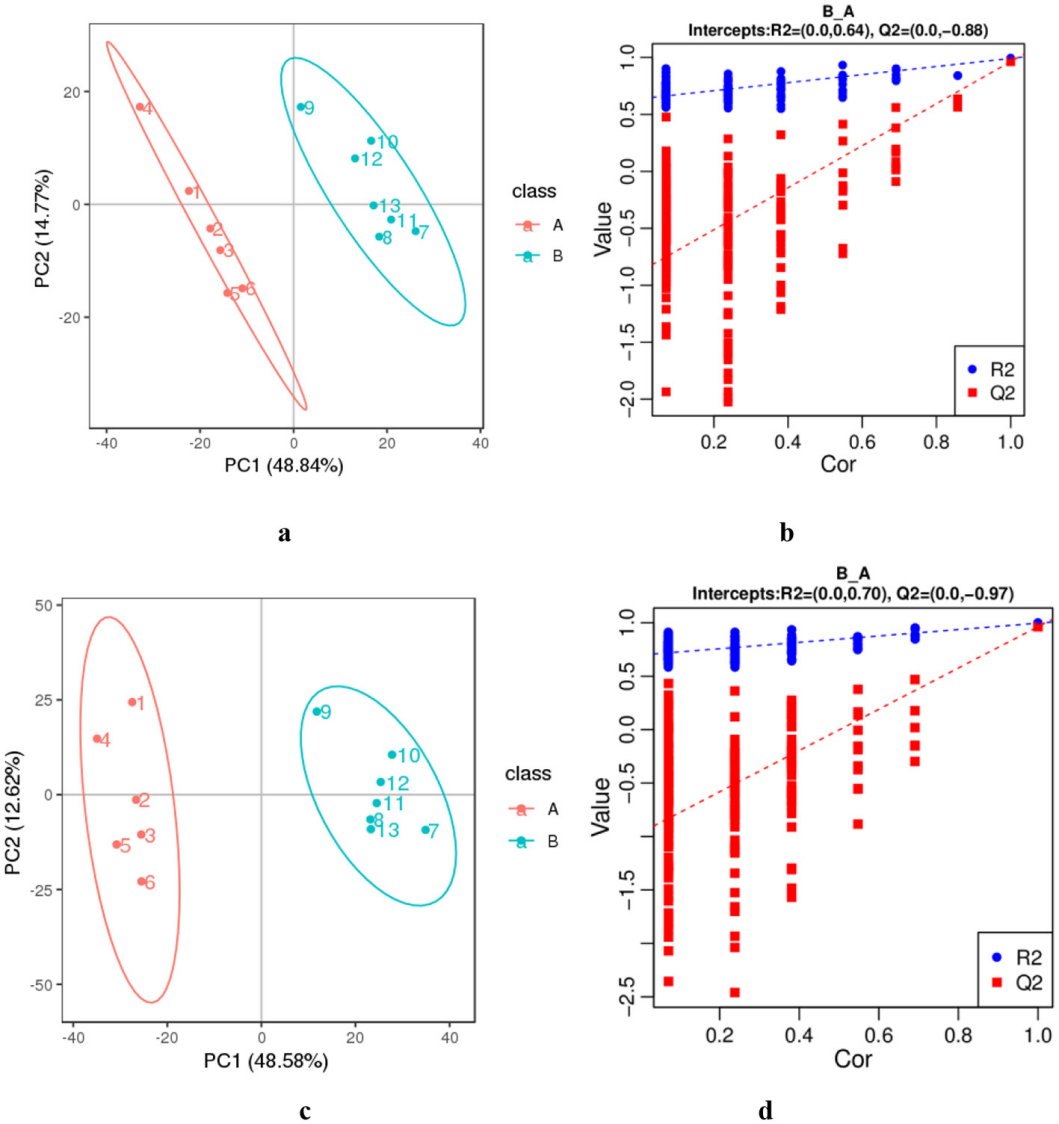
The PCA **(a, c)** and PLS-DA **(b, d)** of lipids from colostrum (A) and mature milk (B). **(a, b)** The negative ion mode. **(c, d)** The positive ion mode. The horizontal axis PC1 and vertical axis PC2 represent the scores of the principal components ranked first and second, respectively. Ellipses represent a 95% confidence interval. The horizontal axis in the validation plots chart represents the correlation between the random group Y and the original group Y, while the vertical axis represents the scores of R2 and Q2.

**Figure 4:**
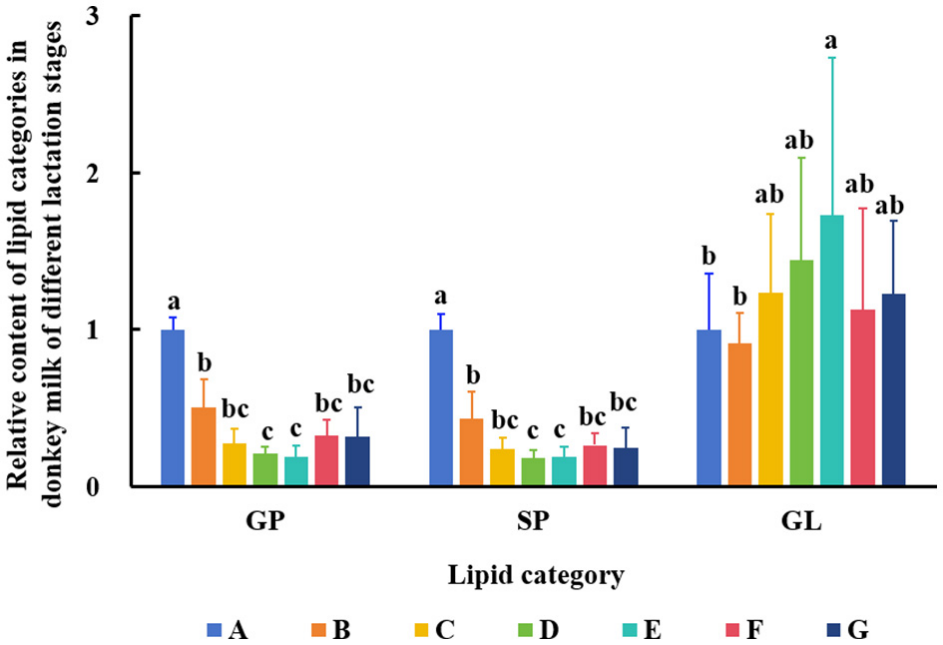
Changes of GP, SP, and GL in DM among different lactation stages. The milk samples were taken from six lactating donkeys at seven different lactation stages (DIM): 1 (A), 30 (B), 60 (C), 90 (D), 120 (E), 150 (F), and 180 (G). The significant differences (*P* < 0.05) were represented in lowercase letters.

The data from DM samples at different lactation stages were analyzed using PCA and PLS-DA. Figure 2 shows the PCA results for all milk samples from 1 to 180 DIM. Figure 3 displays the PCA and PLS-DA score plots of milk lipids from Group A (1 DIM) and Group B (30 DIM). The lactation stage had a significant effect on the DM lipid profile. The contents of GP and SP in colostrum were significantly higher than those in mature milk (*P* < 0.05; Figure 4). Similarly, the relative content of GL in DM at 120 DIM was significantly higher than that at 1 DIM and 30 DIM (*P* < 0.05; Figure 4).

### 3.3 Differential lipid analysis in DM from different lactation stages

**Figure 5:**
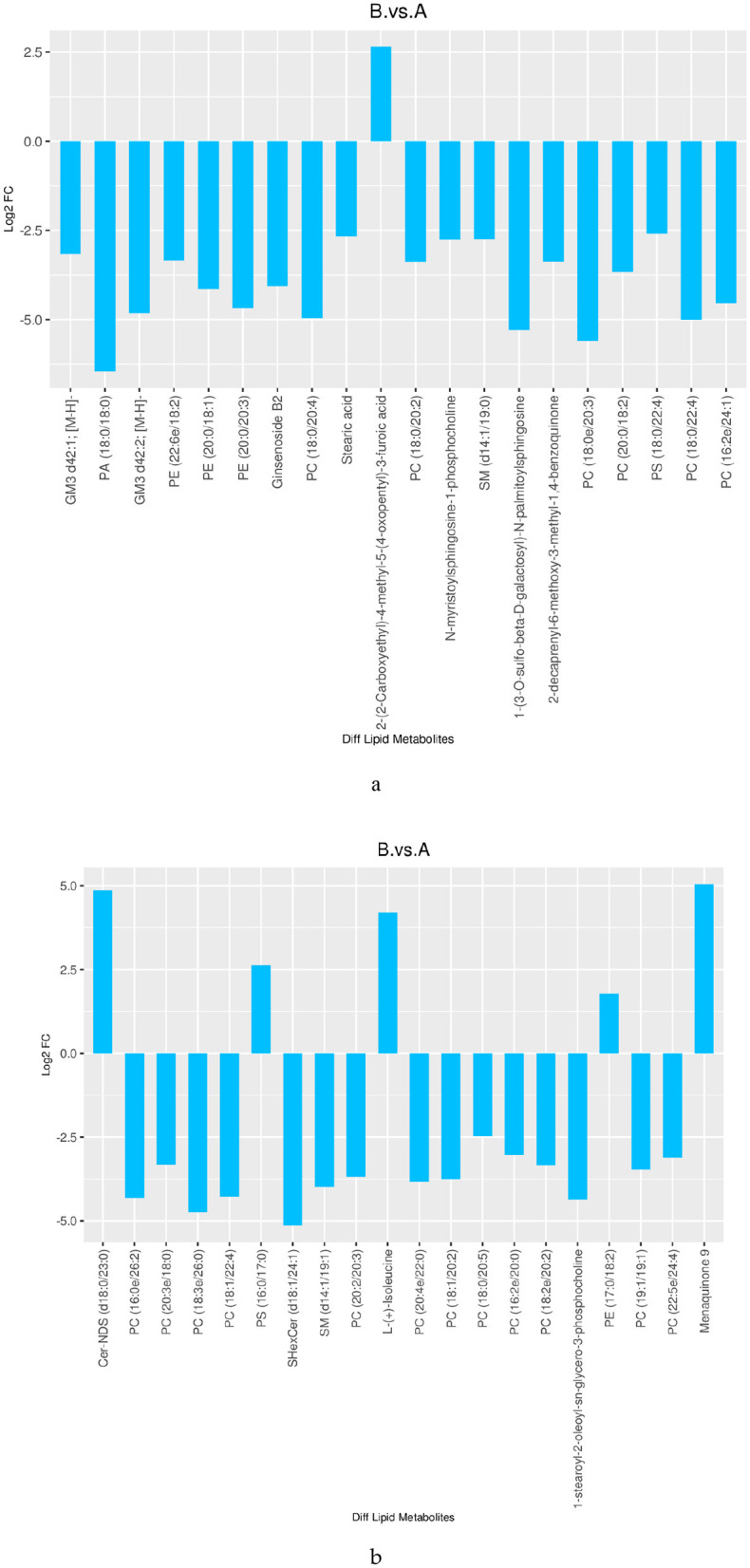
Differences in lipid between mature milk and colostrum (TOP 20) [**(a)** neg; **(b)** pos].

The numbers of differential lipids in DM from different lactation periods are displayed in Table 1 (VIP > 1 and *P* < 0.05). The comparison between mature milk and colostrum (B vs. A) revealed 964 differential compounds, comprising 432 up-regulated and 532 down-regulated lipids. Among the up-regulated lipids, the main categories were 67 types of GPs, 26 types of SPs, and 16 types of GLs. The down-regulated lipids included 248 types of GPs, 135 types of SPs, 11 types of FAs, and 16 types of GLs. Figure 5 shows the top 20 differential lipids in both positive and negative ion modes between mature milk and colostrum, with six lipids increased and 34 lipids decreased.

**Table 1:** Numbers of differential lipids in DM at different lactation stages.

**Compared samples**	**Total identified**	**Differential lipid**	**Increased lipids**	**Decreased lipids**
B vs. A pos	1,610	668	386	282
C vs. A pos		734	351	383
D vs. A pos		737	300	437
E vs. A pos		714	250	464
F vs. A pos		728	382	346
G vs. A pos		724	354	370
B vs. A neg	796	296	46	250
C vs. A neg		349	18	331
D vs. A neg		390	35	355
E vs. A neg		378	20	358
F vs. A neg		350	11	339
G vs. A neg		370	22	348

**Figure 6:**
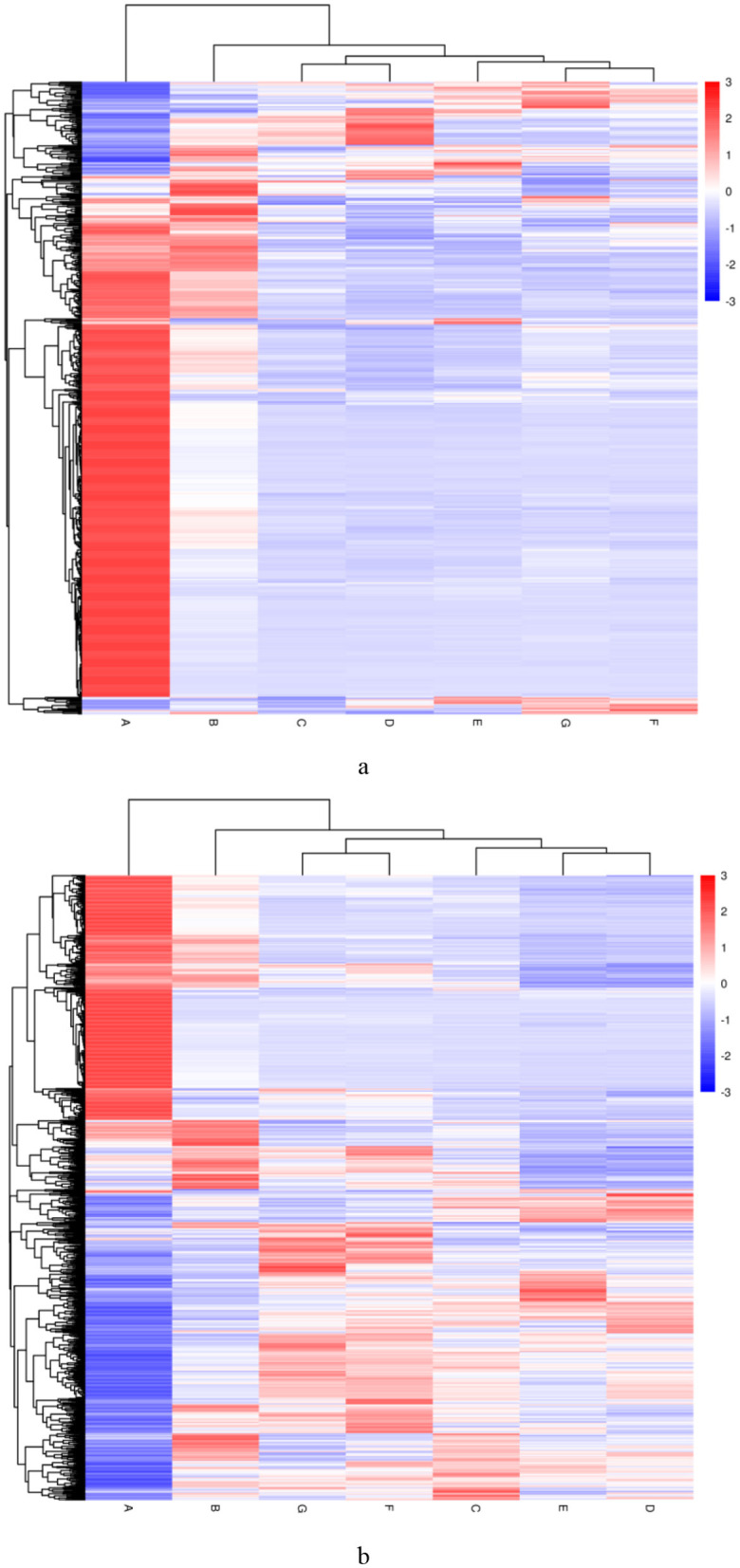
Differential lipid clustering heatmap of DM at different lactation periods. **(a)** The negative and **(b)** positive ion mode. Vertical clustering refers to the clustering of samples, while horizontal clustering refers to the clustering of lipids. The shorter the clustering branch, the higher the similarity. The milk samples were taken from six lactating donkeys at seven different lactation stages (DIM): 1 (A), 30 (B), 60 (C), 90 (D), 120 (E), 150 (F), and 180 (G).

Figure 6 shows the clustering heatmap of differential lipids in DM across different lactation periods (1–180 DIM). Supplementary Table S1 lists the top 20 most significantly differential lipids. Fourteen lipid species were identified as potential biomarkers (VIP > 1 and *P* < 0.05) for distinguishing DM from early, middle, and late lactation (Table 2). Furthermore, four differential lipids were found between colostrum and mid- to late-lactation milk (90–180 DIM), and seven differential lipids were found between colostrum and mature milk (30–180 DIM).

**Table 2:** Differential lipids in DM at different lactation stages compared to colostrum (Top 20).

**Lactation stages**	**Name**	**Formula**	**Molecular weight**	**RT (min)**	**FC^a^**
Early (30–60 DIM)	PC (18:0e/26:4)	C52 H98 N O7 P	879.70952	14.465	0.019792
	PC (o-18:2(9Z,12Z)/24:0)	C50 H98 N O7 P	855.70418	15.168	0.019618
	PC (18:0e/20:3)	C46 H88 N O7 P	843.63649	12.704	0.020683
	1,2-dicapryl-sn-glycero-3-phosphate	C23 H45 O8 P	480.28712	7.108	44.94803
	2-[(5Z,8Z,11Z,14Z,17Z)-eicosapentaenoyl]-sn-glycerol	C23 H36 O4	376.25988	3.405	41.90718
Mid (90–120 DIM)	SM (d14:1/26:1)	C45 H89 N2 O6 P	784.64797	12.675	0.009933
	PC (18:0/19:2)	C45 H86 N O8 P	799.60948	11.853	0.013871
	SM (d25:2/13:0)	C43 H85 N2 O6 P	756.61512	11.249	0.01651
	PC (14:0e/18:1)	C40 H80 N O7 P	717.56822	10.905	0.013091
	N-[(2S,3R,4E,6E)-1,3-Dihydroxy-4,6-tetradecadien-2-yl]icosanamide	C34 H65 N O3	535.49817	7.986	74.58051
Late (150–180 DIM)	GM3 d42:2; [M-H]-	C65 H118 N2 O21	1262.82198	12.538	0.014668
	PC (18:0e/20:3)	C46 H88 N O7 P	843.63649	12.704	0.020298
	PC (19:0/18:2)	C45 H86 N O8 P	799.60977	11.921	0.011914
	PC(P-16:0/16:0)	C40 H80 N O7 P	717.56726	11.653	0.008462
Mid-late (90–180 DIM)	PC (18:3e/22:2)	C48 H88 N O7 P	821.63212	11.76	0.016006
	SM (d28:2/12:1)	C45 H87 N2 O6 P	782.63286	11.763	0.014785
	SM (d25:2/15:1)	C45 H87 N2 O6 P	782.63193	10.9	0.010918
	SM (d14:2/12:0)	C31 H61 N2 O6 P	634.43276	3.582	0.014429
Early-mid-late (30–180 DIM)	SHexCer (d18:1/24:1)	C48 H91 N O11 S	424.30224	12.579	0.018992
	PC (18:0/22:5)	C48 H86 N O8 P	835.60975	11.852	0.017121
	PC (19:0/19:0)	C46 H92 N O8 P	817.67156	13.76	0.015033
	PC (18:0/20:3)	C46 H86 N O8 P	811.60917	11.879	0.011075
	HexCer-NS (d16:1/20:1)	C42 H79 N O8	725.58261	10.336	0.011089
	1-(3-O-sulfo-beta-D-galactosyl)-N-palmitoylsphingosine	C40 H77 N O11 S	779.52292	9.525	0.012098
	PA (18:0/18:0)	C39 H77 O8 P	704.53635	13.597	0.008439

RT, retention time.

^a^FC, fold change, mean value of peak area obtained from DM group/mean value of peak area obtained from DC group.

**Figure 7:**
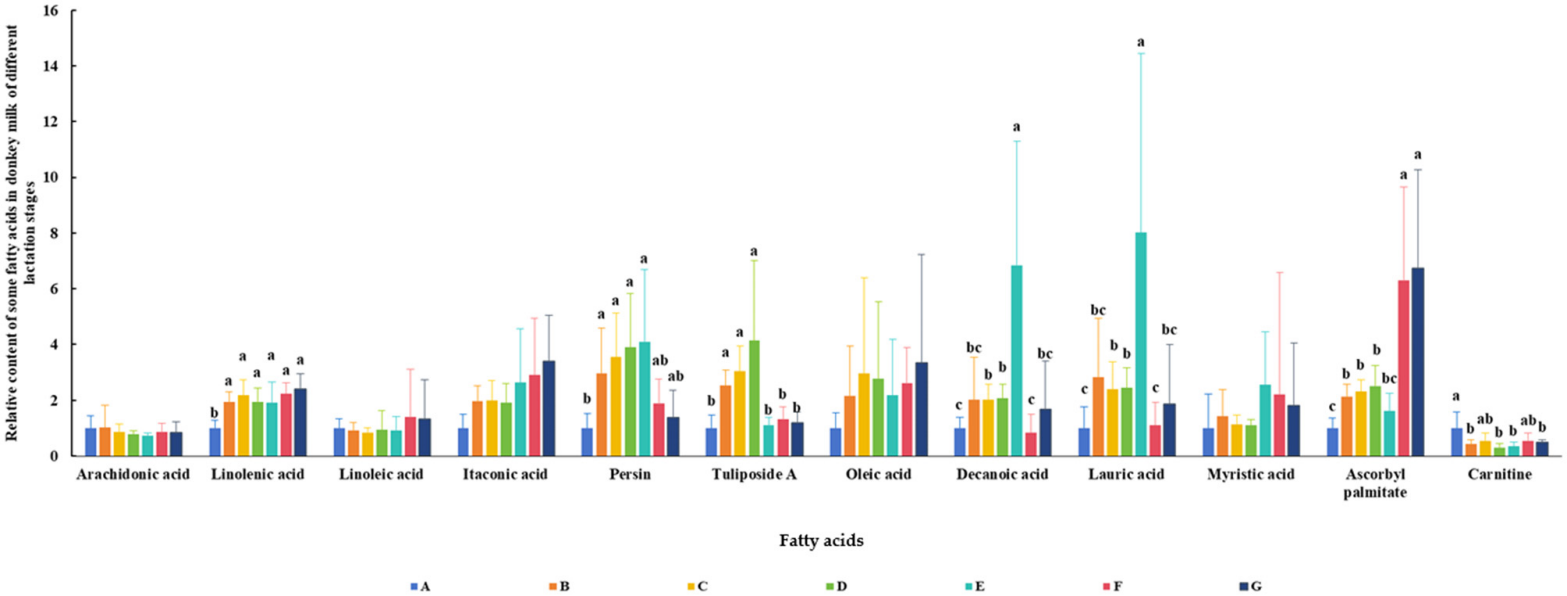
Relative content of some fatty acids in DM of different lactation periods. The milk samples were taken from six lactating donkeys at seven different lactation stages (DIM): 1 (A), 30 (B), 60 (C), 90 (D), 120 (E), 150 (F), and 180 (G). The significant differences (*P* < 0.05) were represented in lowercase letters.

The relative contents of some FAs in DM across different lactation periods are shown in Figure 7. The results revealed that the lactation stage had a significant effect on the levels of α-linolenic acid (ALA), persin, tuliposide A, decanoic acid, lauric acid, ascorbyl palmitate, and carnitine (*P* < 0.05). The changes in persin, tuliposide A, decanoic acid, and lauric acid during lactation were similar, increasing initially and then decreasing (*P* < 0.05). The relative content of ascorbyl palmitate in DM increased with the lactation period and was highest in the late stage (*P* < 0.05). In contrast, the relative content of carnitine was highest in colostrum.

## 4 Discussion

The lipid composition of DM was investigated in this study. A total of 1,552 lipids were detected, spanning five categories and 21 subclasses. GP (62.11%) and SP (22.94%) were the predominant lipids, with PC (27.26%), PE (17.59%), and PS (6.77%) as the major subclasses. In contrast, Li et al. (19) identified 335 lipids from 13 subclasses in DM using UHPLC-QTOF-MS-based quantitative lipidomics, reporting TG, SM, DG, and PE as the main lipids. Another study by Ren et al. (13) detected 1,841 lipids from 29 subclasses using LC-MS, categorized into GL (64.86%), GP (17.71%), SP (8.47%), ST (2.18%), FA (1.14%), and derivatized lipids (5.64%). The dominant subclasses were TG (55.19%), PC (8.69%), DG (8.20%), and PE (5.98%). Li et al. (20) analyzed lipids in donkey colostrum (3 DIM) and mature milk (90 DIM) using LC-MS, identifying 1,774 lipids from six categories and 30 subclasses. TG accounted for 61.95%, followed by DG (7.67%), PC (6.09%), and PE (5.19%). Previous studies have indicated that TG is the most abundant lipid in milk, exceeding 50% in human, donkey, horse, buffalo, cow, goat, sheep, and yak milk (4, 12, 21). Our results differ from these findings, which may be attributed to factors such as animal species and lactation stage. For instance, camel milk contains high levels of PE, PC, and SM (over 30% collectively) (4), while pig milk is rich in PC, SM, and PE (over 53%) (4), aligning more closely with our results. Furthermore, the milk samples in this study included colostrum (1 DIM) and mature milk (30–180 DIM), unlike the studies by Ren et al. (13) and Li et al. (20). The higher fat content and GP proportion in colostrum may explain the discrepancies. Additionally, differences in lipid detection methods across studies could also contribute to the varying results. It should be noted that the composition and content of lipids in DM vary with different diets (22). The changes in lipids in DM may also be caused by dietary differences. Moreover, there were only six lactating donkeys per group in this study, and the individual variations among them may have influenced the results. In future research, it is necessary to increase the sample size and reduce individual differences to improve the generalizability of research results.

The lactation stage had a significant effect on the lipid composition of DM. Li et al. (19) reported 60 differential lipids between donkey mature milk and colostrum, with Hex2Cer, PS, and PI being the main subclasses that differed. In this study, we identified 964 differential lipids between mature milk (30 DIM) and colostrum (1 DIM), of which 432 were increased and 532 were decreased. The GP, SP, GL, and FA categories showed significant differences across lactation periods. Donkey colostrum is rich in GP and SP. These lipids are not only components of cell membranes but also promote infant brain and nerve development and modulate inflammatory responses (23). SM and PC, which are sources of choline, are beneficial for the development of the central nervous system in infants (12, 24). Cer and SM are involved in cell proliferation, differentiation, signal transduction, and the development of the infant immune system (25–27). Therefore, donkey colostrum is an ideal source of nutrition for the optimal growth and development of infants. Conversely, mature donkey milk, especially from the mid-lactation stage (90–120 DIM), contained higher levels of GL. This finding is consistent with the conclusions of Ren et al. (13) and Li et al. (19). Furthermore, 14 types of lipids (5 for early lactation, 5 for mid lactation, and 4 for late lactation) were identified as potential biomarkers for distinguishing DM from different lactation stages.

The composition of FAs in DM is more favorable than that of other milks (16, 28). Santillo et al. (29) revealed that, compared with dairy milk, DM has higher long-chain FA (LC-FA) and lower short-chain FA (SC-FA) contents. In addition, DM has a lower SFA content and is richer in PUFAs than cow milk (28). The PUFA content in DM is similar to that in human milk (4, 30). Donkey milk is rich in PUFAs, such as ALA, linoleic acid (LA), and arachidonic acid (ARA) (4). Similarly, in this study, we detected abundant levels of oleic acid, itaconic acid, decanoic acid, persin, tuliposide A, lauric acid, ascorbyl palmitate, carnitine, myristic acid, ARA, ALA, and LA in DM. Many studies have found that these FAs have many biological activities, such as lipid regulation, antioxidant, anti-inflammatory and immune activation, antibacterial, anti-breast cancer, neuroprotective and anti-apoptotic (31–39).

The content of FAs in DM was affected by the lactation stage. The colostrum was rich in carnitine. Compared with colostrum, mature donkey milk contained more ALA. Milk from early- and mid-lactation contained more persin and tuliposide A. The contents of decanoic acid and lauric acid were highest in mid-lactation milk. Donkey milk at the late lactation stage was rich in ascorbyl palmitate. Carnitine is an essential component for fatty acid metabolism and energy production. After birth, an infant's energy demands for movement, growth, differentiation, and maintaining body temperature increase dramatically, which heavily relies on carnitine-mediated fatty acid oxidation (40). Its high concentrations in colostrum help newborns utilize fat as an energy source. The subsequent decrease in concentration reflects the infant's adaptive physiological adjustments to ongoing digestive system development and changing nutritional demands. Furthermore, by facilitating efficient fat metabolism, carnitine ensures a continuous energy supply to the brain, which is fundamental for neural development (40, 41). As a precursor to docosahexaenoic acid **(**DHA), ALA is the most critical structural fat for infant brain and retinal development (42). Since infants have a limited ability to synthesize DHA endogenously, they rely heavily on ALA provided in breast milk. ALA also possesses anti-inflammatory properties (42, 43). It helps regulate the infant's immune response and prevents excessive inflammation, which is crucial for newborns whose immune systems are not yet fully developed. In addition, ALA can act as pancreatic lipase inhibitors, inhibiting the increase in triglyceride levels in the blood after fat intake, which is beneficial for health and weight management (37). The results of Butt et al. (31) found that persin has an anti-breast cancer effect, which can induce G2-M cell cycle arrest and caspase dependent apoptosis. It has been found that the tuliposides have antibacterial activity (32, 34). Lauric Acid is a well-known antibacterial agent. It also has anti-inflammatory and immune activating effects (35). In study of Sharma et al. (39), the anti-inflammatory, antioxidant, neuroprotective, and anti-apoptotic properties of decanoic acid were observed. The ascorbyl palmitate has antioxidant activities (33). Therefore, DM is an ideal food source for infants and certain patients, as well as a valuable cosmetic ingredient. Specifically, milk from early- and mid-lactation may be more beneficial for patients (e.g., with cancer or enteritis), while DM from mid- and late-lactation is more suitable for use in the cosmetics industry.

In summary, a total of 1,552 lipids were detected in DM, encompassing five lipid categories and 21 subclasses, among which GP and SP were the predominant lipids. The GP, SP, GL, and FA categories varied significantly with the lactation stage. Colostrum was rich in GP and SP, while mature milk contained higher levels of GL. Compared with colostrum, mature donkey milk contained more ALA, whereas colostrum was richer in carnitine. Fourteen lipid species were identified as potential biomarkers for distinguishing DM from different lactation stages. In conclusion, DM contains abundant functional lipids, and the lactation period has a significant effect on its lipid profile. This study contributes to a better understanding of the lipid composition in donkey milk, facilitating its development into high-value nutritional products.

## Data Availability

The data presented in the study are deposited in the Metabolights repository (https://www.ebi.ac.uk/metabolights), accession number MTBLS12948.
