# Supplementary material for: Lipid profile analysis of donkey milk during the lactation
Source: Front Nutr. 2025 Sep 19;12:1662407. doi: 10.3389/fnut.2025.1662407 (PMC12491274; doi:10.3389/fnut.2025.1662407)
Supplement: Supplementary file 1 [file Table_1.doc]

Table S1. Indentification of significantly different lipids between donkey milk from different lactation stages (TOP 20)

|  | Lipid name | Molecular Weight | RT (min) | FC | P value | VIP |
| --- | --- | --- | --- | --- | --- | --- |
| B vs A | PA (18:0/18:0) | 704.5364 | 13.5970 | 0.0114 | 0.0000 | 1.8604 |
|  | HexCer-NS (d16:1/20:1) | 725.5826 | 10.3360 | 0.0189 | 0.0000 | 1.7273 |
|  | PC (18:0e/26:4) | 879.7095 | 14.4650 | 0.0196 | 0.0000 | 2.0534 |
|  | PC (18:0e/20:3) | 843.6365 | 12.7040 | 0.0207 | 0.0000 | 1.9895 |
|  | PC(o-18:2(9Z,12Z)/24:0) | 855.7042 | 15.1680 | 0.0210 | 0.0004 | 1.9421 |
|  | PC (16:0e/15:0) | 705.5674 | 11.4080 | 0.0220 | 0.0000 | 1.8959 |
|  | (3beta,24R)-Ergost-5-en-3-yl 6-O-stearoyl-beta-D-glucopyranoside | 828.6824 | 14.4430 | 0.0237 | 0.0000 | 2.1147 |
|  | PC (19:0/19:0) | 817.6716 | 13.7600 | 0.0254 | 0.0000 | 1.6376 |
|  | 1-(3-O-sulfo-beta-D-galactosyl)-N-palmitoylsphingosine | 779.5229 | 9.5250 | 0.0256 | 0.0000 | 1.4948 |
|  | PC (18:0/20:3) | 811.6092 | 11.8790 | 0.0283 | 0.0000 | 1.4705 |
|  | SHexCer (d18:1/24:1) | 424.3022 | 12.5790 | 0.0286 | 0.0000 | 1.7875 |
|  | PC (18:0/22:5) | 835.6098 | 11.8520 | 0.0303 | 0.0000 | 1.6794 |
|  | PC (18:0/22:4) | 883.6314 | 12.2350 | 0.0312 | 0.0000 | 1.9408 |
|  | PC (18:0/20:4) | 855.6003 | 11.4680 | 0.0321 | 0.0000 | 1.7636 |
|  | (3beta)-Stigmast-5-en-3-yl 6-O-stearoyl-beta-D-glucopyranoside | 842.6977 | 14.7100 | 53.9162 | 0.0000 | 2.0323 |
|  | 1,2-dicapryl-sn-glycero-3-phosphate | 480.2871 | 7.1080 | 40.0059 | 0.0000 | 1.7949 |
|  | Menaquinone 9 | 784.6161 | 12.7980 | 32.8973 | 0.0000 | 2.0032 |
|  | 1-oleoyl-2-linoleoyl-sn-glycero-3-phospho-L-serine | 785.5214 | 11.7930 | 32.6263 | 0.0000 | 1.8877 |
|  | 2-[(5Z,8Z,11Z,14Z,17Z)-eicosapentaenoyl]-sn-glycerol | 376.2599 | 3.4050 | 30.7925 | 0.0000 | 1.5347 |
|  | [(4R)-2-Hydroxy-2-oxido-1,3,2-dioxaphospholan-4-yl]methyl palmitate | 392.2342 | 3.1970 | 29.1865 | 0.0114 | 1.5528 |
| C vs A | PC(P-16:0/16:0) | 717.5673 | 11.6530 | 0.0061 | 0.0021 | 1.5109 |
|  | PA (18:0/18:0) | 704.5364 | 13.5970 | 0.0073 | 0.0000 | 1.5421 |
|  | SM (d25:2/15:1) | 782.6319 | 10.9000 | 0.0094 | 0.0000 | 1.6056 |
|  | HexCer-NS (d16:1/20:1) | 725.5826 | 10.3360 | 0.0094 | 0.0000 | 1.6454 |
|  | PC (18:0/20:3) | 811.6092 | 11.8790 | 0.0104 | 0.0000 | 1.5521 |
|  | 1-(3-O-sulfo-beta-D-galactosyl)-N-palmitoylsphingosine | 779.5229 | 9.5250 | 0.0108 | 0.0000 | 1.4107 |
|  | SM (d14:1/26:1) | 784.6480 | 12.6750 | 0.0121 | 0.0000 | 1.5717 |
|  | SM (d28:2/12:1) | 782.6329 | 11.7630 | 0.0140 | 0.0000 | 1.6555 |
|  | GM3 d42:2; [M-H]- | 1262.8220 | 12.5380 | 0.0142 | 0.0000 | 1.5133 |
|  | PC (19:0/19:0) | 817.6716 | 13.7600 | 0.0148 | 0.0000 | 1.5837 |
|  | PC (18:0/22:5) | 835.6098 | 11.8520 | 0.0161 | 0.0000 | 1.6412 |
|  | PC (18:3e/22:2) | 821.6321 | 11.7600 | 0.0161 | 0.0000 | 1.6224 |
|  | SM (d14:2/12:0) | 634.4328 | 3.5820 | 0.0192 | 0.0005 | 1.2726 |
|  | SHexCer (d18:1/24:1) | 424.3022 | 12.5790 | 0.0192 | 0.0000 | 1.6619 |
|  | PC(o-18:2(9Z,12Z)/24:0) | 855.7042 | 15.1680 | 0.0196 | 0.0004 | 1.6652 |
|  | PC (18:0e/26:4) | 879.7095 | 14.4650 | 0.0200 | 0.0000 | 1.7186 |
|  | PC (18:0/19:2) | 799.6095 | 11.8530 | 0.0201 | 0.0000 | 1.5939 |
|  | PC (18:0e/20:3) | 843.6365 | 12.7040 | 0.0206 | 0.0000 | 1.5165 |
|  | 2-[(5Z,8Z,11Z,14Z,17Z)-eicosapentaenoyl]-sn-glycerol | 376.2599 | 3.4050 | 53.0219 | 0.0000 | 1.51 |
|  | 1,2-dicapryl-sn-glycero-3-phosphate | 480.2871 | 7.1080 | 49.8902 | 0.0000 | 1.54 |
| D vs A | PC (18:0/20:3) | 811.6092 | 11.8790 | 0.0056 | 0.0000 | 1.6289 |
|  | 1-(3-O-sulfo-beta-D-galactosyl)-N-palmitoylsphingosine | 779.5229 | 9.5250 | 0.0077 | 0.0000 | 1.4411 |
|  | PA (18:0/18:0) | 704.5364 | 13.5970 | 0.0078 | 0.0000 | 1.4585 |
|  | PC (19:0/19:0) | 817.6716 | 13.7600 | 0.0078 | 0.0000 | 1.6589 |
|  | SM (d25:2/15:1) | 782.6319 | 10.9000 | 0.0085 | 0.0000 | 1.5447 |
|  | PC(P-16:0/16:0) | 717.5673 | 11.6530 | 0.0088 | 0.0030 | 1.3088 |
|  | SM (d14:1/26:1) | 784.6480 | 12.6750 | 0.0091 | 0.0000 | 1.5794 |
|  | HexCer-NS (d16:1/20:1) | 725.5826 | 10.3360 | 0.0094 | 0.0000 | 1.5629 |
|  | PC (18:0/22:5) | 835.6098 | 11.8520 | 0.0112 | 0.0000 | 1.6730 |
|  | SM (d28:2/12:1) | 782.6329 | 11.7630 | 0.0122 | 0.0000 | 1.6150 |
|  | PC (14:0e/18:1) | 717.5682 | 10.9050 | 0.0125 | 0.0000 | 1.6374 |
|  | PC (18:0/19:2) | 799.6095 | 11.8530 | 0.0134 | 0.0000 | 1.6490 |
|  | 1,2-di-[(9Z,12Z,15Z)-octadecatrienoyl]-sn-glycero-3-phosphocholine | 777.5281 | 9.4710 | 0.0135 | 0.0001 | 1.2098 |
|  | PC (18:3e/22:2) | 821.6321 | 11.7600 | 0.0158 | 0.0000 | 1.5824 |
|  | SHexCer (d18:1/24:1) | 424.3022 | 12.5790 | 0.0160 | 0.0000 | 1.6123 |
|  | SM (d14:2/12:0) | 634.4328 | 3.5820 | 0.0175 | 0.0007 | 1.2296 |
|  | PE (20:0/20:3) | 797.5940 | 11.9340 | 0.0178 | 0.0000 | 1.4649 |
|  | SM (d25:2/13:0) | 756.6151 | 11.2490 | 0.0184 | 0.0000 | 1.4838 |
|  | N-[(2S,3R,4E,6E)-1,3-Dihydroxy-4,6-tetradecadien-2-yl]icosanamide | 535.4982 | 7.9860 | 83.2143 | 0.0000 | 1.4840 |
|  | (2R)-3-(Phosphonooxy)-2-(tetradecanoyloxy)propyl (9Z)-9-tetradecenoate | 590.3947 | 10.4500 | 56.5964 | 0.0009 | 1.1572 |
| E vs A | PC(P-16:0/16:0) | 717.5673 | 11.6530 | 0.0071 | 0.0025 | 1.3591 |
|  | PC (19:0/19:0) | 817.6716 | 13.7600 | 0.0075 | 0.0000 | 1.6545 |
|  | PC (18:0/20:3) | 811.6092 | 11.8790 | 0.0076 | 0.0000 | 1.5641 |
|  | HexCer-NS (d16:1/20:1) | 725.5826 | 10.3360 | 0.0079 | 0.0000 | 1.6148 |
|  | PA (18:0/18:0) | 704.5364 | 13.5970 | 0.0084 | 0.0000 | 1.4718 |
|  | SM (d25:2/15:1) | 782.6319 | 10.9000 | 0.0085 | 0.0000 | 1.5448 |
|  | 1-(3-O-sulfo-beta-D-galactosyl)-N-palmitoylsphingosine | 779.5229 | 9.5250 | 0.0106 | 0.0000 | 1.3978 |
|  | SM (d14:1/26:1) | 784.6480 | 12.6750 | 0.0108 | 0.0000 | 1.5114 |
|  | SM (d14:2/12:0) | 634.4328 | 3.5820 | 0.0119 | 0.0005 | 1.3834 |
|  | PC (18:0/22:5) | 835.6098 | 11.8520 | 0.0124 | 0.0000 | 1.6354 |
|  | SM (d28:2/12:1) | 782.6329 | 11.7630 | 0.0125 | 0.0000 | 1.6015 |
|  | PC (14:0e/18:1) | 717.5682 | 10.9050 | 0.0137 | 0.0000 | 1.5584 |
|  | GM3 d42:2; [M-H]- | 1262.8220 | 12.5380 | 0.0137 | 0.0000 | 1.4885 |
|  | PC (18:0/19:2) | 799.6095 | 11.8530 | 0.0143 | 0.0000 | 1.6201 |
|  | PC (18:3e/22:2) | 821.6321 | 11.7600 | 0.0144 | 0.0000 | 1.5895 |
|  | SM (d25:2/13:0) | 756.6151 | 11.2490 | 0.0146 | 0.0000 | 1.5806 |
|  | SM (d27:2/13:0) | 784.6472 | 12.4140 | 0.0154 | 0.0000 | 1.6902 |
|  | SHexCer (d18:1/24:1) | 424.3022 | 12.5790 | 0.0165 | 0.0000 | 1.6093 |
|  | HexCer-NS (d19:1/24:1) | 823.6949 | 13.7390 | 0.0166 | 0.0132 | 1.2880 |
|  | N-[(2S,3R,4E,6E)-1,3-Dihydroxy-4,6-tetradecadien-2-yl]icosanamide | 535.4982 | 7.9860 | 65.9467 | 0.0000 | 1.2745 |
| F vs A | TAG (16:0-18:1-20:1) | 886.7956 | 17.3440 | 0.0033 | 0.0001 | 1.0208 |
|  | PC (18:0/20:3) | 811.6092 | 11.8790 | 0.0062 | 0.0000 | 1.7039 |
|  | 1-(3-O-sulfo-beta-D-galactosyl)-N-palmitoylsphingosine | 779.5229 | 9.5250 | 0.0063 | 0.0000 | 1.6174 |
|  | PC(P-16:0/16:0) | 717.5673 | 11.6530 | 0.0065 | 0.0025 | 1.4777 |
|  | PA (18:0/18:0) | 704.5364 | 13.5970 | 0.0076 | 0.0000 | 1.5770 |
|  | HexCer-NS (d16:1/20:1) | 725.5826 | 10.3360 | 0.0093 | 0.0000 | 1.6504 |
|  | SM (d25:2/15:1) | 782.6319 | 10.9000 | 0.0117 | 0.0000 | 1.5290 |
|  | GM3 d42:2; [M-H]- | 1262.8220 | 12.5380 | 0.0139 | 0.0000 | 1.5740 |
|  | SM (d14:2/12:0) | 634.4328 | 3.5820 | 0.0142 | 0.0005 | 1.4030 |
|  | PC (18:0/22:5) | 835.6098 | 11.8520 | 0.0158 | 0.0000 | 1.6336 |
|  | SM (d28:2/12:1) | 782.6329 | 11.7630 | 0.0161 | 0.0000 | 1.6016 |
|  | SHexCer (d18:1/24:1) | 424.3022 | 12.5790 | 0.0161 | 0.0000 | 1.7059 |
|  | PC (19:0/18:2) | 799.6098 | 11.9210 | 0.0165 | 0.0000 | 1.5515 |
|  | PC (19:0/19:0) | 817.6716 | 13.7600 | 0.0174 | 0.0000 | 1.5034 |
|  | PC (18:3e/22:2) | 821.6321 | 11.7600 | 0.0179 | 0.0000 | 1.5969 |
|  | PC (18:0/19:2) | 799.6095 | 11.8530 | 0.0191 | 0.0000 | 1.6039 |
|  | PC (18:0e/20:3) | 843.6365 | 12.7040 | 0.0196 | 0.0000 | 1.5850 |
|  | PE (20:0/20:3) | 797.5940 | 11.9340 | 0.0196 | 0.0000 | 1.5534 |
|  | N-[(2S,3R,4E,6E)-1,3-Dihydroxy-4,6-tetradecadien-2-yl]icosanamide | 535.4982 | 7.9860 | 63.9057 | 0.0000 | 1.4573 |
|  | 1-oleoyl-2-linoleoyl-sn-glycero-3-phospho-L-serine | 785.5214 | 11.7930 | 52.1081 | 0.0000 | 1.8395 |
| G vs A | PC (19:0/18:2) | 799.6098 | 11.9210 | 0.0073 | 0.0000 | 1.7003 |
|  | PA (18:0/18:0) | 704.5364 | 13.5970 | 0.0082 | 0.0000 | 1.5361 |
|  | PC (18:0/20:3) | 811.6092 | 11.8790 | 0.0083 | 0.0000 | 1.6170 |
|  | PC(P-16:0/16:0) | 717.5673 | 11.6530 | 0.0104 | 0.0020 | 1.4143 |
|  | HexCer-NS (d16:1/20:1) | 725.5826 | 10.3360 | 0.0115 | 0.0000 | 1.5930 |
|  | 1-(3-O-sulfo-beta-D-galactosyl)-N-palmitoylsphingosine | 779.5229 | 9.5250 | 0.0117 | 0.0000 | 1.4052 |
|  | SM (d14:2/12:0) | 634.4328 | 3.5820 | 0.0141 | 0.0003 | 1.4080 |
|  | SM (d25:2/15:1) | 782.6319 | 10.9000 | 0.0149 | 0.0000 | 1.4714 |
|  | GM3 d42:2; [M-H]- | 1262.8220 | 12.5380 | 0.0154 | 0.0000 | 1.5076 |
|  | PC (18:3e/22:2) | 821.6321 | 11.7600 | 0.0159 | 0.0000 | 1.6087 |
|  | PC (18:0/22:5) | 835.6098 | 11.8520 | 0.0170 | 0.0000 | 1.5990 |
|  | PC (19:0/19:0) | 817.6716 | 13.7600 | 0.0173 | 0.0000 | 1.4841 |
|  | SHexCer (d18:1/24:1) | 424.3022 | 12.5790 | 0.0176 | 0.0000 | 1.6592 |
|  | SM (d28:2/12:1) | 782.6329 | 11.7630 | 0.0183 | 0.0000 | 1.5479 |
|  | PC(o-18:2(9Z,12Z)/24:0) | 855.7042 | 15.1680 | 0.0201 | 0.0004 | 1.6433 |
|  | SM (d28:2/16:1) | 838.6936 | 13.4440 | 0.0205 | 0.0000 | 1.6682 |
|  | 1-stearoyl-2-oleoyl-sn-glycero-3-phosphocholine | 788.6128 | 12.4970 | 0.0208 | 0.0000 | 1.6107 |
|  | PC (18:0e/20:3) | 843.6365 | 12.7040 | 0.0210 | 0.0000 | 1.5462 |
|  | PC (22:5e/13:1) | 749.5465 | 8.6510 | 61.4475 | 0.0069 | 1.2560 |
|  | 2-[(5Z,8Z,11Z,14Z,17Z)-eicosapentaenoyl]-sn-glycerol | 376.2599 | 3.4050 | 52.4243 | 0.0012 | 1.2253 |
